# 4-(4-Nitro­benzene­sulfonamido)pyridinium bromide

**DOI:** 10.1107/S1600536808035265

**Published:** 2008-11-13

**Authors:** Liang Zhao, Qi-Fei Yu, Qiong Wu

**Affiliations:** a School of Food Science, Henan Institute of Science and Technology, Xinxiang 453003, People’s Republic of China; b Department of Food and Biological Engineering, Zhangzhou Institute of Technology, Zhangzhou 363000, People’s Republic of China; c Jilin Key Laboratory for the Biotechnology of Agricultural Products Processing, Changchun University, Changchun 130022, People’s Republic of China

## Abstract

In the title compound, C_11_H_10_N_3_O_4_S^+^·Br^−^, the benzene ring makes an angle of 88.4 (2)° with the pyridinium ring. The dihedral angle between the nitro group and the benzene ring is 16.5 (2)°. The ions in the crystal structure are linked by a combination of inter­molecular N—H⋯Br and non-conventional C—H⋯Br and C—H⋯O hydrogen bonds, forming a three-dimensional network.

## Related literature

For zwitterionic forms of *N*-aryl­benzene­sulfonamides, see: Li *et al.* (2007 ▶); Yu & Li (2007 ▶). For bond-length data, see: Allen *et al.* (1987 ▶). For non-conventional hydrogen bonds, see: Desiraju & Steiner (2001 ▶). For the use of pyridinium derivatives in the construction of supra­molecular architectures, see: Damiano *et al.* (2007 ▶).
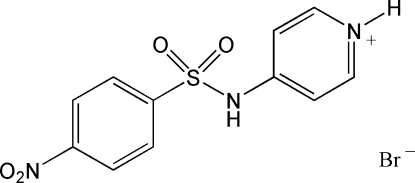


## Experimental

### 

#### Crystal data

C_11_H_10_N_3_O_4_S^+^·Br^−^
*M* *_r_* = 360.19
Monoclinic, 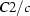

*a* = 38.242 (8) Å
*b* = 5.2852 (11) Å
*c* = 13.941 (3) Å
β = 108.18 (3)°
*V* = 2677.0 (11) Å^3^
*Z* = 8
Mo *K*α radiation
μ = 3.24 mm^−1^
*T* = 113 (2) K
0.10 × 0.04 × 0.02 mm

#### Data collection

Rigaku Saturn CCD area-detector diffractometer
Absorption correction: multi-scan (*CrystalClear*; Rigaku/MSC, 2005 ▶) *T*_min_ = 0.710, *T*_max_ = 0.938
10460 measured reflections
3174 independent reflections
2635 reflections with *I* > 2σ(*I*)
*R*_int_ = 0.050

#### Refinement

*R*[*F* ^2^ > 2σ(*F* ^2^)] = 0.033
*wR*(*F* ^2^) = 0.076
*S* = 1.05
3174 reflections
189 parameters
H atoms treated by a mixture of independent and constrained refinement
Δρ_max_ = 0.68 e Å^−3^
Δρ_min_ = −0.47 e Å^−3^

### 

Data collection: *CrystalClear* (Rigaku/MSC, 2005 ▶); cell refinement: *CrystalClear*; data reduction: *CrystalClear*; program(s) used to solve structure: *SHELXS97* (Sheldrick, 2008 ▶); program(s) used to refine structure: *SHELXL97* (Sheldrick, 2008 ▶); molecular graphics: *SHELXTL* (Sheldrick, 2008 ▶); software used to prepare material for publication: *SHELXTL*.

## Supplementary Material

Crystal structure: contains datablocks global, I. DOI: 10.1107/S1600536808035265/si2122sup1.cif

Structure factors: contains datablocks I. DOI: 10.1107/S1600536808035265/si2122Isup2.hkl

Additional supplementary materials:  crystallographic information; 3D view; checkCIF report

## Figures and Tables

**Table 1:** Hydrogen-bond geometry (Å, °)

*D*—H⋯*A*	*D*—H	H⋯*A*	*D*⋯*A*	*D*—H⋯*A*
N1—H1*A*⋯Br1^i^	0.89 (3)	2.30 (3)	3.195 (2)	173 (3)
N2—H2*A*⋯Br1	0.84 (3)	2.38 (3)	3.225 (3)	174 (2)
C10—H10⋯O3^ii^	0.95	2.44	3.301 (3)	151
C5—H5⋯Br1^iii^	0.95	2.75	3.676 (3)	165

Symmetry codes: (i) 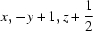; (ii) 
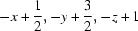
; (iii) 
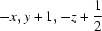
.

**Fig. 1.**
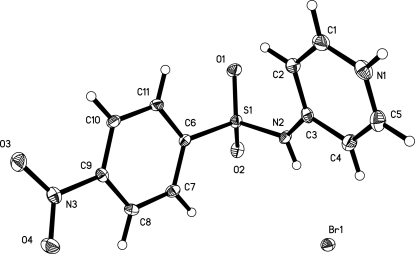
View of one molecule of the title compound showing the atom-labelling scheme. Displacement ellipsoids are drawn at the 35% probability level (arbitrary spheres for the H atoms).

## supplementary crystallographic information

### Comment

Organic pyridinium salts have been widely used in the construction of supramolecular architectures (Damiano *et al.*, 2007). As part of our ongoing studies of supramolecular chemistry involving the pyridinium rings (Li *et al.*, 2007), an X-ray structure analysis of the title compound has been performed. In the cations of the title compound the short C—N distance [N2—C3 = 1.387 (3) Å] has a value between those of a typical C=N double and C—N single bond (1.34–1.38 Å and 1.47–1.50 Å, respectively; Allen *et al.*, 1987). This might be indicative of a slight conjugation of the sulphonamide π electrons N with those of the pyridinium ring. The benzene ring makes an angle of 88.4 (2) ° with the pyridinium ring. The dihedral angle between the nitro group and the benzene ring is 163.5 (2) °. The S atom has a tetrahedral geometry and the Br anion link the cationic molecule into chains along the *c* axis. The ions in the crystal structure are linked by a combination of intermolecular N—H···Br and non-conventional C—H···Br and C—H···O hydrogen bonds (Table 1) to form a three-dimensional network (Desiraju & Steiner, 2001).

### Experimental

A solution of 4-nitrobenzenesulfonyl chloride (2.2 g, 10 mmol) in CH_2_Cl_2_ (10 ml) was added dropwise to a suspension of 4-aminopyridine (0.9 g, 10 mmol) in CH_2_Cl_2_ (10 ml) at room temperature with stirring. The reaction mixture was stirred overnight. The yellow solid obtained was washed with warm water to obtain the title compound in a yield of 60.6%. A colorless single-crystal suitable for X-ray analysis was obtained by slow evaporation of an hydrobromic acid (5%) solution at room temperature over a period of a week. Analysis calculated for C_11_H_10_N_3_O_4_SBr: C 36.68, H 2.80, N 11.67%; found: C 36.70, H 2.52, N 11.98%.

### Refinement

The N-bound H atoms were located in a difference map and their coordinates were refined with *U*_iso_(H) = 1.2*U*_eq_(N). The C-bound H atoms were positioned geometrically (C—H =0.95 Å) and refined as riding with *U*_iso_(H) = 1.2*U*_eq_(C).

### Crystal data

**Table:**

C_11_H_10_N_3_O_4_S^+^·Br^–^	*F*_000_ = 1440
*M**_r_* = 360.19	*D*_x_ = 1.787 Mg m^−^^3^
Monoclinic, *C*2/*c*	Mo *K*α radiation λ = 0.71073 Å
Hall symbol: -C 2yc	Cell parameters from 3479 reflections
*a* = 38.242 (8) Å	θ = 2.2–27.9º
*b* = 5.2852 (11) Å	µ = 3.24 mm^−^^1^
*c* = 13.941 (3) Å	*T* = 113 (2) K
β = 108.18 (3)º	Needle, colorless
*V* = 2677.0 (11) Å^3^	0.10 × 0.04 × 0.02 mm
*Z* = 8	

### Data collection

**Table:**

Rigaku Saturn CCD area-detector diffractometer	3174 independent reflections
Radiation source: rotating anode	2635 reflections with *I* > 2σ(*I*)
Monochromator: confocal	*R*_int_ = 0.050
Detector resolution: 7.31 pixels mm^-1^	θ_max_ = 27.9º
*T* = 113(2) K	θ_min_ = 2.2º
ω and φ scans	*h* = −45→50
Absorption correction: multi-scan(CrystalClear; Rigaku/MSC, 2005)	*k* = −6→4
*T*_min_ = 0.710, *T*_max_ = 0.938	*l* = −18→18
10460 measured reflections	

### Refinement

**Table:**

Refinement on *F*^2^	Secondary atom site location: difference Fourier map
Least-squares matrix: full	Hydrogen site location: inferred from neighbouring sites
*R*[*F*^2^ > 2σ(*F*^2^)] = 0.033	H atoms treated by a mixture of independent and constrained refinement
*wR*(*F*^2^) = 0.076	*w* = 1/[σ^2^(*F*_o_^2^) + (0.0332*P*)^2^] where *P* = (*F*_o_^2^ + 2*F*_c_^2^)/3
*S* = 1.05	(Δ/σ)_max_ = 0.001
3174 reflections	Δρ_max_ = 0.68 e Å^−^^3^
189 parameters	Δρ_min_ = −0.47 e Å^−^^3^
Primary atom site location: structure-invariant direct methods	Extinction correction: none

### Special details

**Table:**

Geometry. All e.s.d.'s (except the e.s.d. in the dihedral angle between two l.s. planes) are estimated using the full covariance matrix. The cell e.s.d.'s are taken into account individually in the estimation of e.s.d.'s in distances, angles and torsion angles; correlations between e.s.d.'s in cell parameters are only used when they are defined by crystal symmetry. An approximate (isotropic) treatment of cell e.s.d.'s is used for estimating e.s.d.'s involving l.s. planes.
Refinement. Refinement of *F*^2^ against ALL reflections. The weighted *R*-factor *wR* and goodness of fit *S* are based on *F*^2^, conventional *R*-factors *R* are based on *F*, with *F* set to zero for negative *F*^2^. The threshold expression of *F*^2^ > σ(*F*^2^) is used only for calculating *R*-factors(gt) *etc*. and is not relevant to the choice of reflections for refinement. *R*-factors based on *F*^2^ are statistically about twice as large as those based on *F*, and *R*- factors based on ALL data will be even larger.

### Fractional atomic coordinates and isotropic or equivalent isotropic displacement parameters (Å^2^)

**Table:**

	*x*	*y*	*z*	*U*_iso_*/*U*_eq_	
Br1	0.038458 (6)	−0.03831 (5)	0.156635 (19)	0.02228 (9)	
S1	0.134013 (15)	−0.12886 (11)	0.40908 (5)	0.01823 (14)	
O1	0.14776 (4)	−0.1752 (3)	0.51529 (13)	0.0248 (4)	
O2	0.12760 (4)	−0.3323 (3)	0.33820 (13)	0.0240 (4)	
O3	0.25567 (5)	0.7285 (4)	0.36991 (13)	0.0304 (4)	
O4	0.21939 (5)	0.7239 (3)	0.21551 (13)	0.0277 (4)	
N1	0.05972 (6)	0.5912 (4)	0.53090 (18)	0.0279 (5)	
N2	0.09433 (5)	0.0162 (4)	0.38299 (17)	0.0191 (4)	
N3	0.22900 (5)	0.6463 (4)	0.30319 (16)	0.0207 (4)	
C1	0.09132 (7)	0.4746 (5)	0.5789 (2)	0.0271 (6)	
H1	0.1046	0.5230	0.6460	0.033*	
C2	0.10479 (6)	0.2877 (5)	0.53330 (18)	0.0227 (5)	
H2	0.1277	0.2101	0.5672	0.027*	
C3	0.08437 (6)	0.2113 (5)	0.43571 (18)	0.0196 (5)	
C4	0.05109 (6)	0.3348 (5)	0.38843 (19)	0.0261 (6)	
H4	0.0365	0.2863	0.3224	0.031*	
C5	0.03964 (7)	0.5254 (5)	0.4376 (2)	0.0304 (6)	
H5	0.0173	0.6115	0.4053	0.036*	
C6	0.16392 (6)	0.0923 (4)	0.37923 (18)	0.0165 (5)	
C7	0.16066 (6)	0.1306 (5)	0.27787 (18)	0.0192 (5)	
H7	0.1436	0.0342	0.2268	0.023*	
C8	0.18247 (6)	0.3099 (5)	0.25237 (17)	0.0186 (5)	
H8	0.1808	0.3389	0.1838	0.022*	
C9	0.20687 (6)	0.4465 (4)	0.32927 (18)	0.0164 (5)	
C10	0.21101 (6)	0.4056 (5)	0.43050 (18)	0.0188 (5)	
H10	0.2285	0.4995	0.4815	0.023*	
C11	0.18912 (6)	0.2252 (5)	0.45572 (17)	0.0193 (5)	
H11	0.1914	0.1931	0.5244	0.023*	
H1A	0.0529 (8)	0.723 (6)	0.561 (2)	0.040 (8)*	
H2A	0.0799 (8)	−0.010 (5)	0.324 (2)	0.018 (7)*	

### Atomic displacement parameters (Å^2^)

**Table:**

	*U*^11^	*U*^22^	*U*^33^	*U*^12^	*U*^13^	*U*^23^
Br1	0.02226 (14)	0.02479 (15)	0.01857 (15)	−0.00186 (10)	0.00461 (11)	−0.00007 (10)
S1	0.0162 (3)	0.0167 (3)	0.0210 (3)	0.0022 (2)	0.0046 (2)	0.0039 (2)
O1	0.0228 (8)	0.0270 (10)	0.0216 (10)	0.0012 (8)	0.0025 (7)	0.0111 (8)
O2	0.0219 (8)	0.0179 (9)	0.0323 (10)	0.0006 (7)	0.0085 (8)	−0.0030 (8)
O3	0.0290 (9)	0.0333 (11)	0.0296 (11)	−0.0136 (8)	0.0099 (8)	−0.0083 (9)
O4	0.0351 (10)	0.0260 (10)	0.0230 (10)	−0.0022 (8)	0.0105 (8)	0.0055 (8)
N1	0.0323 (12)	0.0243 (12)	0.0317 (14)	−0.0043 (10)	0.0170 (11)	−0.0065 (10)
N2	0.0139 (10)	0.0224 (11)	0.0182 (12)	0.0011 (8)	0.0010 (9)	−0.0021 (9)
N3	0.0230 (10)	0.0178 (10)	0.0248 (12)	0.0004 (9)	0.0124 (9)	−0.0026 (9)
C1	0.0280 (14)	0.0316 (15)	0.0234 (15)	−0.0087 (11)	0.0104 (12)	−0.0043 (12)
C2	0.0209 (12)	0.0261 (13)	0.0209 (13)	−0.0024 (10)	0.0063 (11)	0.0011 (11)
C3	0.0186 (11)	0.0182 (12)	0.0250 (13)	−0.0050 (10)	0.0109 (10)	−0.0001 (10)
C4	0.0203 (12)	0.0310 (15)	0.0256 (15)	0.0033 (11)	0.0052 (11)	−0.0009 (12)
C5	0.0246 (13)	0.0292 (15)	0.0398 (17)	0.0059 (11)	0.0136 (13)	0.0012 (13)
C6	0.0137 (10)	0.0182 (12)	0.0163 (12)	0.0017 (9)	0.0029 (9)	0.0013 (9)
C7	0.0204 (11)	0.0185 (12)	0.0162 (13)	0.0008 (10)	0.0022 (10)	−0.0038 (10)
C8	0.0226 (11)	0.0208 (12)	0.0129 (11)	0.0003 (10)	0.0063 (10)	−0.0005 (10)
C9	0.0172 (11)	0.0146 (11)	0.0194 (13)	0.0029 (9)	0.0087 (10)	0.0011 (10)
C10	0.0160 (11)	0.0232 (12)	0.0149 (12)	0.0011 (9)	0.0015 (10)	−0.0018 (10)
C11	0.0175 (11)	0.0246 (13)	0.0146 (12)	0.0042 (10)	0.0032 (9)	0.0037 (10)

### Geometric parameters (Å, °)

**Table:**

S1—O2	1.4288 (18)	C2—H2	0.9500
S1—O1	1.4292 (18)	C3—C4	1.399 (3)
S1—N2	1.637 (2)	C4—C5	1.366 (4)
S1—C6	1.773 (2)	C4—H4	0.9500
O3—N3	1.226 (3)	C5—H5	0.9500
O4—N3	1.232 (3)	C6—C11	1.385 (3)
N1—C5	1.333 (4)	C6—C7	1.394 (3)
N1—C1	1.335 (4)	C7—C8	1.380 (3)
N1—H1A	0.89 (3)	C7—H7	0.9500
N2—C3	1.387 (3)	C8—C9	1.385 (3)
N2—H2A	0.84 (3)	C8—H8	0.9500
N3—C9	1.468 (3)	C9—C10	1.387 (3)
C1—C2	1.359 (3)	C10—C11	1.385 (3)
C1—H1	0.9500	C10—H10	0.9500
C2—C3	1.400 (3)	C11—H11	0.9500
			
O2—S1—O1	121.05 (11)	C5—C4—C3	119.6 (3)
O2—S1—N2	104.58 (11)	C5—C4—H4	120.2
O1—S1—N2	109.04 (11)	C3—C4—H4	120.2
O2—S1—C6	108.52 (10)	N1—C5—C4	120.3 (2)
O1—S1—C6	107.48 (11)	N1—C5—H5	119.8
N2—S1—C6	105.09 (11)	C4—C5—H5	119.8
C5—N1—C1	121.6 (2)	C11—C6—C7	121.8 (2)
C5—N1—H1A	120.0 (19)	C11—C6—S1	119.90 (17)
C1—N1—H1A	118.3 (19)	C7—C6—S1	118.27 (18)
C3—N2—S1	128.28 (19)	C8—C7—C6	119.4 (2)
C3—N2—H2A	115.5 (17)	C8—C7—H7	120.3
S1—N2—H2A	114.9 (18)	C6—C7—H7	120.3
O3—N3—O4	123.7 (2)	C7—C8—C9	118.3 (2)
O3—N3—C9	118.3 (2)	C7—C8—H8	120.9
O4—N3—C9	117.9 (2)	C9—C8—H8	120.9
N1—C1—C2	121.1 (3)	C8—C9—C10	122.8 (2)
N1—C1—H1	119.4	C8—C9—N3	119.0 (2)
C2—C1—H1	119.4	C10—C9—N3	118.2 (2)
C1—C2—C3	119.1 (2)	C11—C10—C9	118.7 (2)
C1—C2—H2	120.5	C11—C10—H10	120.7
C3—C2—H2	120.5	C9—C10—H10	120.7
N2—C3—C4	117.2 (2)	C10—C11—C6	119.0 (2)
N2—C3—C2	124.6 (2)	C10—C11—H11	120.5
C4—C3—C2	118.2 (2)	C6—C11—H11	120.5
			
O2—S1—N2—C3	172.9 (2)	O1—S1—C6—C7	167.55 (17)
O1—S1—N2—C3	42.1 (2)	N2—S1—C6—C7	−76.4 (2)
C6—S1—N2—C3	−72.9 (2)	C11—C6—C7—C8	−1.6 (3)
C5—N1—C1—C2	1.7 (4)	S1—C6—C7—C8	177.10 (17)
N1—C1—C2—C3	−2.3 (4)	C6—C7—C8—C9	−0.2 (3)
S1—N2—C3—C4	168.56 (19)	C7—C8—C9—C10	1.9 (3)
S1—N2—C3—C2	−13.2 (3)	C7—C8—C9—N3	−177.11 (19)
C1—C2—C3—N2	−177.1 (2)	O3—N3—C9—C8	−164.7 (2)
C1—C2—C3—C4	1.1 (3)	O4—N3—C9—C8	16.1 (3)
N2—C3—C4—C5	178.9 (2)	O3—N3—C9—C10	16.2 (3)
C2—C3—C4—C5	0.5 (4)	O4—N3—C9—C10	−162.90 (19)
C1—N1—C5—C4	0.0 (4)	C8—C9—C10—C11	−1.8 (3)
C3—C4—C5—N1	−1.1 (4)	N3—C9—C10—C11	177.23 (19)
O2—S1—C6—C11	−146.30 (18)	C9—C10—C11—C6	0.0 (3)
O1—S1—C6—C11	−13.8 (2)	C7—C6—C11—C10	1.7 (3)
N2—S1—C6—C11	102.3 (2)	S1—C6—C11—C10	−176.96 (17)
O2—S1—C6—C7	35.0 (2)		

### Hydrogen-bond geometry (Å, °)

**Table:**

*D*—H···*A*	*D*—H	H···*A*	*D*···*A*	*D*—H···*A*
N1—H1A···Br1^i^	0.89 (3)	2.30 (3)	3.195 (2)	173 (3)
N2—H2A···Br1	0.84 (3)	2.38 (3)	3.225 (3)	174 (2)
C10—H10···O3^ii^	0.95	2.44	3.301 (3)	151
C5—H5···Br1^iii^	0.95	2.75	3.676 (3)	165

Symmetry codes: (i) *x*, −*y*+1, *z*+1/2; (ii) −*x*+1/2, −*y*+3/2, −*z*+1; (iii) −*x*, *y*+1, −*z*+1/2.
